# Success with EASE: Who benefits from a STEM learning community?

**DOI:** 10.1371/journal.pone.0213827

**Published:** 2019-03-22

**Authors:** Sabrina Solanki, Peter McPartlan, Di Xu, Brian K. Sato

**Affiliations:** 1 School of Education, University of California, Irvine, United States of America; 2 Department of Molecular Biology and Biochemistry, University of California, Irvine, United States of America; Universitat Luzern, SWITZERLAND

## Abstract

During the past few decades, there has been a nationwide push to improve performance and persistence outcomes for STEM undergraduates. As part of this effort, recent research has emphasized the need for focus on not only improving the delivery of course content, but also addressing the social-psychological needs of students. One promising intervention type that has been proposed as a multifaceted way to address both cognitive and social-psychological aspects of the learning process is the learning community. Learning communities provide students with opportunities to build a strong support system in college and are generally associated with increased student engagement and integration with campus systems and cultures. In this study, we examine the impact of a learning community intervention for first-year biological sciences majors, the Enhanced Academic Success Experience (EASE) program. Incoming freshmen are assigned to EASE based on their SAT (or ACT equivalent) Math score, a metric demonstrated to be a key predictor of student success in the program. We find that enrollment in EASE is correlated with higher STEM course grades; an increase of 0.25 (on a 0–4 point scale) in cumulative first-year GPA; and gains in non-academic outcomes, such as measures of sense of belonging and academic integration. Further, these outcomes are more pronounced for particular subgroup populations. For example, whereas surveyed male students seemed to benefit academically from participating in a learning community, female students reported a greater sense of belonging in regard to the biological sciences major and reported higher values for behavioral indicators of academic integration. Lastly, we find that the EASE program is positively correlated with students’ *intention* to stay in the biological sciences major. And, among the three race-oriented groups, this impact is most pronounced for under-represented students. In light of these findings, we discuss the potential of discipline-specific learning community programs to improve academic outcomes for students most at risk of leaving STEM majors, such as students underprepared for college level coursework.

## Introduction

A considerable number of studies in science, technology, engineering, and mathematics (STEM) education research fields have focused on improving outcomes for undergraduate students using two intervention types: interventions that impact content delivery both inside and outside the classroom and those that address social-psychological aspects of the student experience. Examples of interventions include the incorporation of active learning strategies [1–3], the structuring of at-home activities that help students prepare for class [4–5], and values-affirmation writing exercises [6–7]. Interventions of this nature have been developed in response to nationwide concern regarding the low persistence of STEM undergraduates in their academic majors—an issue that disproportionately impacts underrepresented minority (URM) students, low-income students, and first-generation college-going students [8].

Another tactic used to improve STEM outcomes has been to alter the college student experience at the program or institution-wide level. Examples of interventions utilizing this model include summer bridge programs, structured independent research experiences, and STEM learning centers [9–13]. In the same vein are learning community programs, the focus of the present study. Learning communities are intentionally designed to increase opportunities for students to interact with peers, faculty, and the curriculum, which allows for the construction of a strong support system. A number of studies have found a positive correlation between participation in a learning community and traditional academic markers of success, also finding positive outcomes for students most at risk for leaving college, such as students underprepared for college-level coursework [14–15]. Unlike the majority of studies about learning communities, the present study is unique in that it examines whether a learning community can be particularly beneficial within a specific discipline—the biological sciences—and therefore has the potential to contribute to the small but growing body of work on learning communities in STEM education.

### Learning communities

Learning communities, at its core, promote peer-to-peer and student-faculty interaction and provide students with a number of opportunities to build a strong support system. The majority of learning communities incorporate active and collaborative learning activities (e.g., students co-enrolling in courses) and promote involvement in complementary academic and social activities that extend beyond the classroom (e.g., students meet weekly in a study skills course and/or with a group mentor). Faculty involved in learning communities are encouraged to use active pedagogical strategies that foster meaningful interaction between students and instructors. They are also encouraged to engage with one another and think about ways to support student learning outcomes [15]. All of these components build institutionalized social support networks that subsequently buttress academic support systems [15–26].

The positive impacts of learning communities such as the Meyerhoff Scholars Program [27] and Posse Foundation programs [28] exemplify the important role social integration plays in academic success, which long-standing theories of college persistence have espoused [29–30]. As documented in literature from the past two decades, a common measure of students’ social integration is sense of belonging, which is based on perceived social support, connectedness, and mattering [31]. The ways in which sense of belonging is associated with persistence have become increasingly apparent, with research detailing that it is positively linked to students’ motivation [31–33], engagement [34], and achievement [35]. Thus, it has become clear that the potential of learning communities to further students’ sense of belonging is one of the defining components of this intervention type [36].

#### Extant literature

Prior research has documented that first-year students who participate in learning communities have higher grades, retention rates, and self-reported levels of engagement than their peers who have not had a learning community experience. Further, learning community students report studying more with peers outside of class and becoming more involved in academic activities [19; 22–26].

Zhao and Kuh [26], for example, used the National Survey of Student Engagement—a survey widely used to assess the quality of the undergraduate college experience—to estimate the impact of learning communities on a myriad of student outcomes. Learning community participation was positively associated with a number of outcomes related to student engagement, such as academic effort, academic integration, and collaborative learning. Learning community participants were also more likely to interact with faculty members. Lastly, students in learning communities reported being more satisfied with their college experience as compared to students who did not participate in learning communities. For student outcomes, effect sizes ranged from 0.23 to 0.60. Thus, even though the authors note limitations to the study, the moderate correlations documented in their paper substantiate the idea that learning communities are a powerful support structure that can impact the overall student experience in college.

The most rigorous evaluation of learning communities to date comes from a recent report by MDRC [37]. Using random assignment, MDRC evaluated the impacts of a one-semester learning community program on students assigned to developmental English classes at six different community colleges. In general, the study failed to find any consistent evidence that learning communities positively influenced students’ college persistence and academic performance.

A number of plausible reasons could explain these null effects. Students participating in the learning community programs this study assessed came from a variety of fields. The lack of common interests and goals represented could have substantially weakened the connections between students and sense of belonging, a major component of learning communities that is often cited as being highly correlated to student academic progress and retention decisions. Also, all six programs’ interventions lasted only one semester, and programs included only one component of a learning community: paired courses.

It is important to note, however, that the MRDC study makes a unique contribution to the literature about learning communities, as it is the only learning community study to use the gold standard in research design: randomization of participants for treatment and control conditions. Findings can therefore be viewed as having a causal interpretation, which is important because they therefore shed light on the possibility that correlational studies, most of which have shown positive impacts, could be over-stating the benefits of participating in a learning community program. The implications of using correlational research design in relation to the present study is discussed in the last section of this paper.

Most published learning community studies were implemented for the general population of first-year college students or for those in developmental education programs, such as MDRC [37]. Only a small number were implemented in STEM programs. The nature of STEM programs, however, makes students well-positioned to reap the benefits of a learning community. This is because students in STEM programs often face discouragement and a loss of confidence due to initially low grades; they experience the weakening of morale as a result of competitive STEM culture and the generally unwelcoming atmosphere of STEM courses. Further, students are often overwhelmed by STEM’s rigorous curriculum, fast-paced instruction, demand for independent work, and content overload in courses taught by often unengaging STEM faculty [38,39]. In contrast, inclusive learning communities provide academic support and are headed by faculty interested in effective instruction and strong student-instructor relationships, indicating that these communities can foster positive student development in STEM.

The few studies about learning communities in STEM education show positive impacts. In Dagley et al. [18], for example, researchers evaluated the EXCEL program, a first-year learning community for STEM students at the University of Central Florida. In addition to offering traditional learning community components, EXCEL gives participants the option to live together in on-campus residential housing, which appears to be beneficial since, in this particular context, a number of social interactions brought faculty and students to the residential space to engage in informal activities.

When Dagley et al. [18] compared the learning outcomes of EXCEL participants to those of a comparison group of students who had declared a STEM major and had the same standardized test math score range, they found that first-year retention, long-term retention, and graduation rates were higher for the EXCEL cohorts than for the comparison group. Specifically, retention of students in a STEM major was 43% higher for program participants than for the comparison group. Further, female, African-American, and Hispanic individuals in the program were correlated with higher retention and graduation rates than similar comparison students.

### The present study

In this study, we evaluate the Enhanced Academic Success Experience (EASE) program, a learning communities program implemented in the school of biological sciences at a large university in the Western United States, the University of California, Irvine. Specifically, we explore whether the benefits learning communities offer at the college level and for first-year students can have similar impact when a learning community is instituted within a specific field of study, such as biological sciences. Within learning community literature, only a few programs have been implemented and evaluated in STEM fields, as noted earlier. However, these studies have shown that learning communities can play an important role in fostering an early sense of engagement and institutional identification [40], which can be especially important as students face the challenges inherent in STEM courses of study [41].

We also focus our efforts on evaluating the ways in which the EASE program impacts certain subgroups. For example, learning communities may especially affect the academic performance and persistence of underrepresented populations in college, such as first-generation college students. First-generation college students have parents who have not attained a four-year college degree and often come from families with fewer financial resources, in addition to having attended lower quality high schools than their continuing-generation peers. As a result, many begin their college career requiring additional academic support and are uncertain about how to successfully navigate the college experience [42]. The support system a learning community provides therefore has the potential to reduce socioeconomic achievement gaps in college.

Learning communities also provide students with an environment that fosters feelings of belonging. Indeed, upon their arrival in college underrepresented populations are prone to feeling a lack of belongingness, which is an important component of social integration and predictor of persistence [31,36,43]. Cultural Mismatch Theory has offered an explanation for why underrepresented populations, such as first-generation students, are more prone to experience this phenomenon. First proposed by Stephens, Fryberg, Markus, Johnson, and Covarrubias [44], Cultural Mismatch Theory illuminates the stark contrast between the community-oriented values typical of first-generation students and the values of the university environment. Specifically, it purports that individual performance is contingent upon whether people experience a match or a mismatch between their own cultural norms and the norms that are institutionalized in a given setting. First-generation and low-income students often come from working-class communities that value interdependence and attention paid to others, yet higher education culture emphasizes independence and competition [38,44]. This contrast is especially evident in competitive STEM disciplines and can make first-generation students especially likely to perform poorly and drop out. First-generation URM students must also combat additional belongingness issues, as they are likely to react negatively when encountering challenges in college, interpreting them as evidence that they do not belong at their institution or within their major [45]. In promoting social support, learning communities may be capable of improving belongingness for underrepresented groups [36].

#### The EASE program

The EASE program at the University of California, Irvine follows a learning communities model; it is a multi-faceted program that aims to improve freshman students’ performance and persistence in biological sciences. Eligibility is determined by the Department of Biological Sciences during the summer prior to the start of the school year. Specifically, all incoming freshmen with SAT (or ACT equivalent) Math scores of lower than 600 are enrolled in the EASE program. Administrators in the Biological Sciences department assign students to cohorts (roughly 25 students per cohort group for a total of sixteen cohorts) and adjust schedules accordingly so that student groups are enrolled in the same course section for all first-year STEM courses. EASE students are made aware of their EASE status at the summer Student-Parent Orientation Program (SPOP).

EASE provides a number of ways for students to become integrated into campus culture and campus systems, both academically and socially [29–30]. Specifically, students in the EASE program are provided the following resources:

1. Academic remediation: EASE students are required to take an additional developmental chemistry course online the summer prior to college matriculation. This course is designed to prepare biological sciences majors for college-level courses in chemistry and biology.
2. Academic and social support: Each cohort is enrolled in the same biology and chemistry courses (lectures and discussion sections) for one year. The majority of EASE students co-enrolled in 5 courses during their first year, one of which was a preparatory general chemistry course during the first academic quarter and was required in order to proceed with the rest of the general chemistry curriculum. The main goal of co-enrollment is for cohorts to engage in learning activities and develop strong relationships, which is hoped to increase students’ sense of belonging in the biological sciences department and at the institution. Each cohort is also matched with a senior biological sciences mentor. Mentors are upperclassman biological sciences majors selected by the department; they have a tutoring background and have excelled in introductory biological sciences courses. The mentors provide increased academic support and serve as students’ main guide to all campus resources and opportunities. Lastly, EASE students participate in a weekly 50-minute seminar led by an EASE mentor. Seminar topics are generally academic in nature and focus particularly on study skills and metacognition. General first-year issues are also discussed, in addition to advice about how to navigate the first-year experience. The personalized advising and guidance students receive through the EASE program is intended to not only fill students’ knowledge gaps, but also provide support as students develop academic and social-emotional skills during their first year in college.

In analyzing the EASE program, we expected social integration within the biological sciences major to manifest itself in two particular ways. First, in keeping with other studies’ finding that learning communities impact students’ sense of institution-specific belonging [36, 21], we expected learning communities to improve students’ sense of belonging within the biological sciences major, thereby strengthening students’ belief that they fit the subject and that their involvement is valued [36]. Second, as students often enter college with academic and social concerns about the challenges they will face [33], we anticipated that the social support systems in EASE would assuage these concerns, including those about being socially ostracized and being judged for poor performance.

Evidence of the program’s effect on students’ academic integration was expected to manifest itself in distinct ways as well. First, we anticipated that students would engage more in course-related behaviors, such as visiting faculty during office hours, participating in student study groups, and using other campus resources. Because the social support features of learning communities are designed to encourage academic collaboration, we hypothesized that we would find evidence of improved academic integration in interactions with teachers, advisors, and study groups [46]. We also expected that the program’s supplemental instruction and advising would support students’ interest in the biological sciences, with academic interest serving as an especially strong predictor of persistence among undergraduate students [47–48]. Consequently, we predicted that furthered course-related behaviors and interest in the biological sciences major would correspond with higher grades in students’ biological science courses.

**Fig 1:**
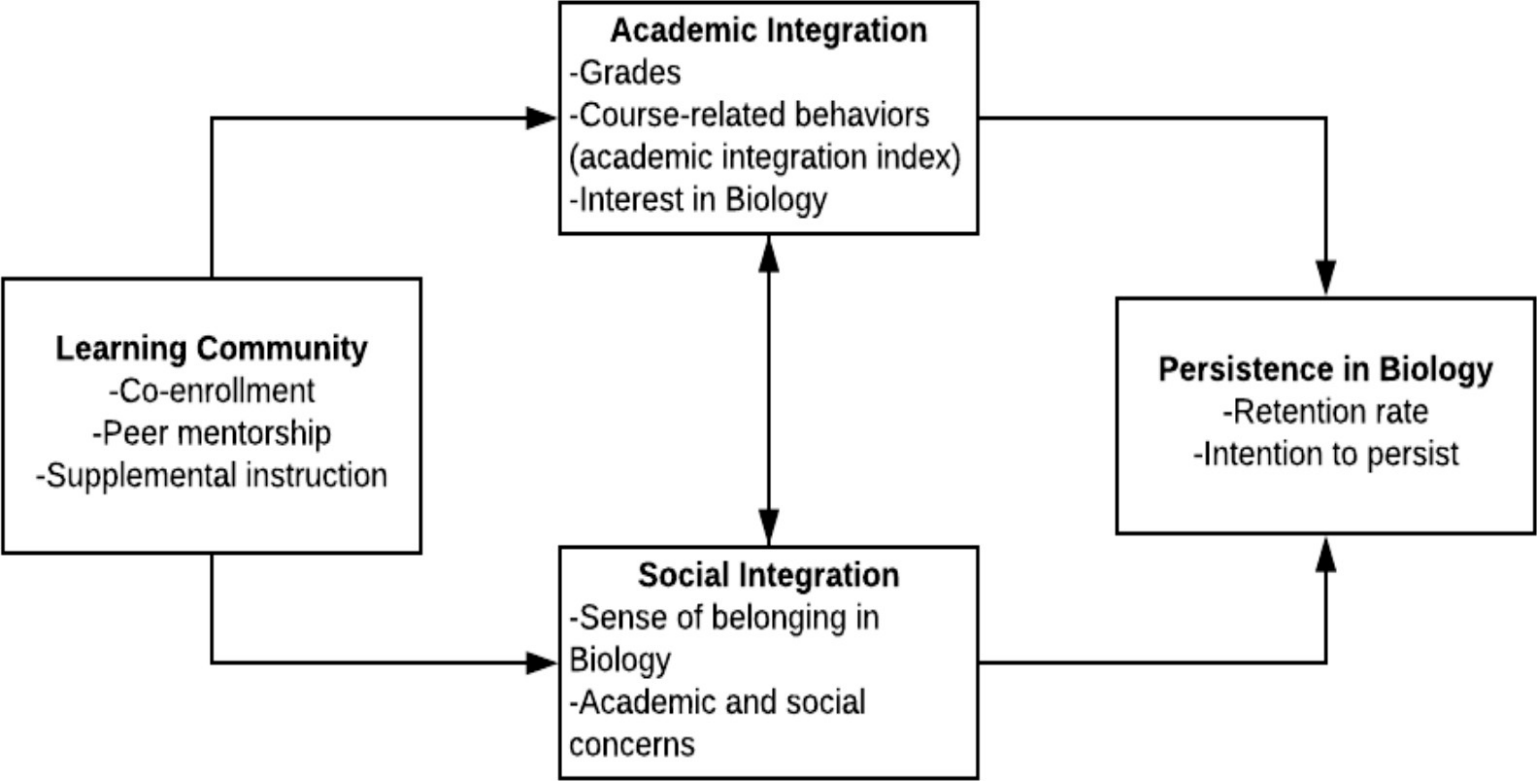
Conceptual model of EASE learning community program.

The conceptual model in Fig 1 depicts the hypothesized mechanisms through which EASE was expected to impact persistence. The model is based on popular theories of college persistence that emphasize the complementary roles of academic integration and social integration; the measures used in the present study are listed as indicators of academic and social integration. It is important to note that the present study measures only the total association of the learning community, with academic and social integration functioning acting as independent entities. However, as the vertical arrows in the model convey, we believe both that persistence is generated through academic and social integration and that these factors are mutually reinforcing. For instance, a greater sense of belonging within the biological sciences major (social integration) would be expected to enhance a student’s interest in biology (academic integration) [35]. By the same token, students who get higher grades (academic integration) as a result of EASE’s supplementary instruction would be expected to have fewer academic and social concerns (social integration) [49].

In this study, we address the following research questions:

1. Is enrollment in the EASE program correlated with improved academic outcomes?
2. Is enrollment in the EASE program correlated with social-psychological measures of the student experience?
3. Are students in the EASE program more likely to remain in the biological sciences major than students not enrolled in the program?
4. Do the impacts of the EASE program vary by student subgroup?

## Methods

### Study context

This study took place at the University of California, Irvine, a public research-intensive university located in the Western United States. It focuses on first-year students in the biological sciences (Bio Sci) major. This study was performed with approval from the University of California, Irvine Institutional Review Board (HS# 2015–2310).

The EASE group consisted of 42.7% (N = 388) of first-year biological sciences majors, grouped into sixteen cohorts. Descriptive data regarding the students who were and were not placed in the EASE program can be found in Table 1. As shown in Table 1, female students were more likely to get placed in the EASE program. The EASE program also included a larger proportion of students traditionally marginalized in college: 55% of participants belong to a URM group, 63.4% of participants are first-generation college students, and 54.6% come from low-income family households. Lastly, EASE participants earned much lower average SAT-reading and SAT-math scores.

**Table 1:** Demographic data for study population.

	Full Student Sample		EASE—No		EASE—Yes		
	Mean (%)	SD	Mean (%)	SD	Mean (%)	SD	P value
Female	0.684	(0.465)	0.613	(0.488)	0.780	(0.415)	0.000
White	0.125	(0.331)	0.131	(0.338)	0.116	(0.321)	0.518
URM	0.337	(0.473)	0.177	(0.381)	0.550	(0.498)	0.000
Asian	0.539	(0.499)	0.692	(0.462)	0.333	(0.472)	0.000
First-gen status	0.484	(0.500)	0.372	(0.484)	0.634	(0.482)	0.000
Low-income status	0.407	(0.492)	0.303	(0.460)	0.546	(0.498)	0.000
SAT Reading score (mean)	558.9	(76.45)	581.8	(77.42)	528.2	(63.33)	0.000
SAT Math score (mean)	598.0	(88.48)	653.4	(70.54)	523.9	(45.35)	0.000
N	907		519		388		

**Table 1**. Demographic data of Bio Sci majors, including those that are and are not in the EASE program. URM consists of Hispanic, African-American, and Native-American students. Differences between students in and not in the EASE program were determined using t-tests with the P value indicated.

### Data collected

Data collection took a variety of forms, including an online survey instrument implemented at the beginning and end of the fall quarter and a slightly modified version presented at the end of the spring quarter. These surveys measured a variety of student attitudes and behaviors regarding the field of biology, such as sense of belonging, academic and social concerns, academic integration, and interest. All three surveys are provided in the supporting information (S1 File). We briefly describe each construct below.

*Belonging in biology* assesses the extent to which students feel they belong in the discipline of biology at UCI. Items were adapted from Hoffman’s [36] Sense of Belonging Scale to ensure they are specific to the biology discipline instead of to the university in general. This measure includes 8 items; students were asked to indicate how true statements, such as “I have developed personal relationships with other students in my Bio Sci classes” and “I feel comfortable seeking help from my Bio Sci teachers before or after class,” were true on a 1 (not at all true) to 7 (very true) Likert scale. Cronbach’s alpha for the 8 items is equal to 0.85.

*Academic and social concerns* conveys the extent to which participants worry that other students will dislike them or unfairly evaluate their academic ability [49]. This measure includes 3 items—for example, “In college, I sometimes worry that people will dislike me.” Students were asked to respond using the same Likert scale discussed above. Cronbach’s alpha for the three items is equal to 0.73.

*Academic integration* indicates the frequency with which participants engaged in various school-related activities—such as talking to faculty, planning with academic advisors, and attending study groups—during their first term on campus. This measure included 5 items. Cronbach’s alpha is equal to 0.60.

*Interest in biology* is a measure inspired by the Eccles’ [50] Expectancy-Value Model of motivation, which is a critical means of evaluating how much students value a field of study. Individual items were adapted from Harackiewicz’s [51] and include, for example, “I think the field of Biology is very interesting.” This measure included 3 items. Cronbach’s alpha for the 3 items is equal to 0.91.

Student demographic data—including gender, ethnicity, first-generation status, low-income status, SAT Reading score, and SAT (or ACT equivalent) Math score—was collected from the campus registrar. We also collected student outcome data that includes information about course performance in two first-year biology courses: Bio Sci 93, an introductory course that covers biology basics, and Bio Sci 94, the follow-up course. Data regarding first-year overall GPA and retention within the biological sciences major at the end of the first year was also collected and included in the analysis as student outcome measures.

### Data analysis

To explore the relationship between the EASE program and student academic and nonacademic outcomes, we use an ordinary least squares (OLS) estimation strategy

$$\Upsilon_{i}=\beta_{0}+\beta_{1}({EASE}_{i})+{Χ}_{i}+\mu_{i}$$ (1)

in which EASE is the key explanatory variable and is equal to 1 if the student participated in the program; Χ_*i*_ includes demographic characteristics (e.g., gender, race, first-generation status, and low-income status) and academic preparedness characteristics (e.g., SAT section scores). μ_i_ is the error term.

Additionally, we explore whether gaps in academic achievement and in the social-psychological measures are wider or narrower for certain student subgroups. For this aspect of analysis, we include an interaction term between a given individual attribute (such as ‘female’) and EASE status in Eq 1. We present the formal equation below. For all analyses, robust standard errors were used.

$$\Upsilon_{i}=\beta_{0}+\beta_{1}({EASE}_{i})+\beta_{2}({Attribute}_{i})+\beta_{3}({EASE}_{i}*{Attribute}_{i})+{Χ}_{i}+\mu_{i}$$ (2)

We report the β_1_ coefficient, which indicates the impact of EASE on the reference group (for example, male students), and the β_3_ coefficient, which indicates whether EASE reduced the academic achievement gap (or in the case of gender, the gender achievement gap). We run a separate regression conditioning on the student attributes using Eq (1). Where noted, missing values have been adjusted using a dummy variable approach [52].

## Results

### Impact of EASE on student performance outcomes

We first wanted to identify whether participation in the EASE program is correlated with improved student outcomes. Table 2 provides the results for a number of these academic outcomes, including performance in freshman biology courses, first-year cumulative GPA, and retention within the biological sciences major for students who were and were not in the EASE program. When controlling for a variety of demographic characteristics, we find that enrollment in the EASE program is correlated with significantly higher grades in Bio Sci 94–0.38 grade points higher on a 0–4 point scale—and a 0.24 boost in first-year GPA.

**Table 2:** Estimates for the impact of EASE on performance outcomes.

	Bio Sci 93 course grade	Bio Sci 94 course grade	Year 1 GPA	Retained
EASE	-0.055	0.380***	0.242***	0.013
	(0.085)	(0.088)	(0.057)	(0.026)
Female	-0.047	-0.144**	-0.005	-0.032**
	(0.065)	(0.063)	(0.044)	(0.015)
URM	-0.153	-0.225**	-0.163***	-0.043
	(0.094)	(0.088)	(0.063)	(0.031)
Asian	-0.185**	-0.202**	-0.142**	0.021
	(0.087)	(0.080)	(0.059)	(0.025)
First-gen status	0.020	-0.037	-0.029	0.004
	(0.066)	(0.070)	(0.046)	(0.017)
Low-income status	-0.027	0.010	-0.003	0.022
	(0.067)	(0.070)	(0.046)	(0.018)
SAT Reading score	0.003***	0.003***	0.002***	0.000***
	(0.000)	(0.000)	(0.000)	(0.000)
SAT Math score	0.003***	0.003***	0.002***	0.000
	(0.001)	(0.001)	(0.000)	(0.000)
N	903	853	839	899

**Table 2**. Robust standard errors in included in parentheses. The reference group White. Course grade and GPA estimates use a 0–4 point scale. Missing values have been adjusted using a dummy variable approach.

* p < 0.10

** p < 0.05

*** p < 0.01.

As discussed earlier, the purpose of our study is to better understand whether certain student subgroups (e.g., URM students, female students, and first-generation students) benefit particularly from the learning community experience, with the results of our analysis found in Table 3. Using gender as an example, both male and female EASE students earned higher Bio Sci 94 grades than their non-EASE counterparts did (column 2). Specifically, male EASE students and female EASE students earned grades 0.53 and 0.34 grade points higher (on a 0–4 point scale), respectively, than their non-EASE counterparts. However, the β_3_ coefficient, EASE*Female, is not significant. Thus, the gender achievement gap in the EASE sample is not significantly different than that in the non-EASE sample.

**Table 3:** Estimates for the impact of EASE on performance outcomes for the full sample and for student subgroups.

		(1)		(2)		(3)		(4)	
		Biology 93 course Grade		Biology 94 course Grade		Year 1 GPA		Retained	
Gender									
Full-sample estimates									
	EASE (Male)	-0.113	(0.169)	0.525	(0.168)***	0.339	(0.114)***	-0.037	(0.034)
	EASE*Female	0.111	(0.196)	-0.167	(0.199)	-0.114	(0.133)	0.077	(0.048)
Subsample estimates									
	Male (N = 276)	-0.113	(0.170)	0.525	(0.169)***	0.339	(0.115)***	-0.037	(0.035)
	Female (N = 602)	-0.002	(0.099)	0.357	(0.106)***	0.225	(0.067)***	0.040	(0.034)
Race									
Full-sample estimates									
	EASE (White)	-0.014	(0.215)	0.475	(0.181)***	0.367	(0.132)***	-0.001	(0.071)
	EASE*URM	0.114	(0.248)	-0.070	(0.233)	-0.089	(0.160)	0.066	(0.086)
	EASE*Asian	-0.121	(0.252)	-0.093	(0.227)	-0.141	(0.159)	-0.008	(0.077)
Subsample estimates									
	White (N = 110)	-0.014	(0.220)	0.475	(0.185)**	0.367	(0.135)***	-0.001	(0.073)
	URM (N = 297)	0.100	(0.124)	0.405	(0.147)***	0.278	(0.091)***	0.066	(0.049)
	Asian (N = 471)	-0.135	(0.131)	0.382	(0.137)***	0.226	(0.088)**	-0.009	(0.030)
First-generation status									
Full-sample estimates									
	EASE	0.104	(0.119)	0.593	(0.135)***	0.334	(0.088)***	0.043	(0.041)
	EASE*First-gen status	-0.228	(0.173)	-0.330	(0.182)*	-0.121	(0.118)	-0.039	(0.054)
Subsample estimates									
	Continuing-gen status (N = 446)	0.104	(0.119)	0.593	(0.135)***	0.334	(0.088)***	0.043	(0.041)
	First-gen status (N = 432)	-0.124	(0.125)	0.263	(0.122)**	0.214	(0.078)***	0.005	(0.035)
Low-income status									
Full-sample estimates									
	EASE	0.125	(0.103)	0.601	(0.117)***	0.318	(0.076)***	0.072	(0.037)*
	EASE*Low-income status	-0.339	(0.172)**	-0.435	(0.180)**	-0.135	(0.118)	-0.113	(0.051)**
Subsample estimates									
	Low-income status = 0 (N = 514)	0.125	(0.103)	0.601	(0.117)***	0.318	(0.076)***	0.072	(0.037)*
	Low-income status = 1 (N = 364)	-0.214	(0.137)	0.167	(0.136)	0.183	(0.090)**	-0.041	(0.035)

**Table 3**. Robust standard errors in included in parentheses. All models include the following student controls: female, URM, Asian, first-generation status, low-income status, SAT Reading score, SAT Math score. The reference group is White. Course grade and GPA estimates are reported using a 0–4 point scale. Missing values have been adjusted using a dummy variable approach.

* p < 0.10

** p < 0.05

*** p < 0.01.

In terms of race, all three student subgroup populations benefited from the EASE program. White students, URM students, and Asian students earned Bio Sci 94 grades that were 0.48, 0.41, and 0.38 grade points higher, respectively, than those of their non-EASE counterparts. Again, we do not find a significant interaction term (EASE*URM) to indicate that the racial achievement gap decreases given EASE involvement. Overall, whereas groups of students traditionally less-represented in STEM (e.g., female, URM, first generation, and low-income) saw gains in Bio Sci 94 grades and first-year cumulative GPA, these gains are not greater than those observed for their non-at-risk counterparts (Table 3).

### Impact of EASE on social-psychological measures of the student experience

Learning communities are considered a way to not only alleviate academic issues common among at-risk students, but also further students’ social psychological well-being, positively affecting their sense of belonging, academic and social concerns, academic integration, and interest in science. As shown in Table 4, we find that student involvement in EASE is correlated with statistically significant higher levels of sense of belonging and academic integration at the end of students’ first term in college. Specifically, EASE students reported values for sense of belonging and academic integration that were 0.21 and 0.32 standard deviation units larger, respectively, than those reported by non-EASE students.

**Table 4:** Estimates for the impact of EASE on social-psychological outcomes of the student experience.

	(1)	(2)	(3)	(4)
	Sense of Belonging	Academic & Social Concerns	Academic Integration	Academic Interest
EASE	0.206**	0.077	0.322***	-0.057
	(0.080)	(0.072)	(0.110)	(0.083)
Female	0.043	0.055	-0.052	-0.020
	(0.063)	(0.053)	(0.076)	(0.067)
URM	-0.109	-0.048	-0.107	0.075
	(0.105)	(0.082)	(0.130)	(0.109)
Asian	-0.046	0.056	-0.130	0.011
	(0.094)	(0.074)	(0.113)	(0.096)
First-gen status	0.054	-0.106*	-0.093	0.018
	(0.067)	(0.059)	(0.080)	(0.067)
Low-income status	0.027	-0.000	-0.023	0.117*
	(0.060)	(0.057)	(0.078)	(0.065)
SAT Reading score	-0.000	-0.000	-0.002***	0.002***
	(0.000)	(0.000)	(0.001)	(0.001)
SAT Math score	0.002***	0.000	0.001	-0.000
	(0.000)	(0.000)	(0.001)	(0.001)
N	832	834	864	829

**Table 4.** Robust standard errors in included in parentheses. Dummy variable approach to missing values used. All items measured at the end of the fall quarter and standardized to have a mean of 0 and a standard deviation of 1. All models include a pre-score. For Academic and Social Concerns, higher values indicate more concern.

* p < 0.10

** p < 0.05

*** p < 0.01.

Similar to our efforts to analyze academic outcomes, we explore the possibility of heterogeneous treatment effects. As shown in Table 5, we find significant differences in values regarding sense of belonging reported by female students in EASE and female students not in EASE. Specifically, female EASE students reported values for the sense of belonging measure that were 0.18 standard deviation units higher than the values reported by female students not in EASE. This impact is even more pronounced for the measure of academic integration, with female EASE students reporting values that were 0.44 standard deviation units larger than those reported by non-EASE female students. Additionally, for the measure of academic integration, the interaction term EASE*Female is positive and marginally significant, indicating that the large increase for female students is responsible for a gender gap that favors female students.

**Table 5:** Estimates for the impact of EASE on social-psychological outcome measures of the student experience for student subgroups.

			(1)		(2)		(3)		(4)	
			Sense of Belonging		Academic and Social Concerns		Academic Integration		Academic Interest	
Gender										
	Full-sample estimates									
		EASE (Male)	0.243	(0.179)	0.280	(0.140)**	0.003	(0.201)	-0.011	(0.164)
		EASE*Female	-0.066	(0.200)	-0.258	(0.164)	0.435	(0.242)*	-0.043	(0.191)
	Subsample estimates									
		Male (N = 251)	0.243	(0.180)	0.280	(0.141)**	0.003	(0.202)	-0.011	(0.165)
		Female (N = 558)	0.177	(0.089)**	0.023	(0.085)	0.437	(0.134)***	-0.054	(0.097)
Race										
	Full-sample estimates									
		EASE (White)	0.145	(0.227)	0.262	(0.184)	0.436	(0.300)	0.043	(0.242)
		EASE*URM	-0.047	(0.259)	-0.243	(0.224)	-0.297	(0.363)	0.073	(0.279)
		EASE*Asian	0.171	(0.257)	-0.174	(0.214)	-0.004	(0.332)	-0.220	(0.270)
	Subsample estimates									
		White (N = 100)	0.145	(0.233)	0.262	(0.189)	0.436	(0.307)	0.043	(0.249)
		URM (N = 269)	0.098	(0.125)	0.019	(0.128)	0.139	(0.203)	0.115	(0.137)
		Asian (N = 440)	0.316	(0.120)***	0.088	(0.109)	0.431	(0.141)***	-0.178	(0.117)
First-generation status										
	Full-sample estimates									
		EASE	0.308	(0.133)**	-0.032	(0.111)	0.210	(0.160)	-0.005	(0.132)
		EASE*First-gen status	-0.222	(0.168)	0.223	(0.153)	0.202	(0.222)	-0.053	(0.172)
	Subsample estimates									
		Cont’ing-gen status (N = 410)	0.308	(0.133)**	-0.032	(0.111)	0.210	(0.160)	-0.005	(0.132)
		First-gen status (N = 399)	0.086	(0.103)	0.190	(0.105)*	0.412	(0.154)***	-0.059	(0.110)
Low-income (LI) status										
	Full-sample estimates									
		EASE	0.304	(0.116)***	-0.009	(0.101)	0.181	(0.147)	-0.088	(0.117)
		EASE*Low-income status	-0.276	(0.161)*	-0.435	(0.180)**	0.301	(0.222)	0.090	(0.168)
	Subsample estimates									
		LI status = 0 (N = 477)	0.304	(0.116)***	-0.009	(0.101)	0.181	(0.147)	-0.088	(0.117)
		LI status = 1 (N = 332)	0.028	(0.112)	0.203	(0.110)*	0.483	(0.167)***	0.002	(0.120)

**Table 5**. Robust standard errors in included in parentheses. All models include the following student controls: female, URM, Asian, first-generation status, low-income status, SAT Reading score, SAT Math score. The reference group is White. All items measured at the end of fall quarter and standardized to have a mean of 0 and a standard deviation of 1. All models include a pre-score. For Academic and Social Concerns, higher values indicate more concern. Missing values have been adjusted using a dummy variable approach.

* p < 0.10

** p < 0.05

*** p < 0.01.

Male students reported social-psychological outcome measure values larger than those reported by their non-EASE counterparts only for the measure of academic and social concerns. The positive coefficient, however, indicates that male EASE students reported having more concerns than their non-EASE counterparts.

Of the three different race groups, Asian students seemed most affected by EASE. Specifically, the coefficients indicate that Asian students in EASE experienced a greater sense of belonging (β_1_ = 0.32) and engaged in behaviors that indicated they were more academically integrated within the major (β_1_ = 0.43). EASE did not have similarly significant effects for White and URM students. Lastly, first-generation students in EASE reported being more concerned about their academic ability than their non-EASE counterparts. However, these same students reported much higher values for the item measuring academic integration (β_1_ = 0.41). Low-income students in EASE also reported much higher values for the academic integration measure than their non-EASE counterparts (β_1_ = 0.48).

### Impact of EASE on the desire to remain in the biological sciences major

The EASE program had little correlation with increases in major retention, as measured by the number of students still declared as majoring in Bio Sci by the end of their freshman year. This may be due, in part, to how uncommon it is for a student to be removed from a major in the freshman year, as it is a multi-step process involving a probationary period that may have prevented us from observing departure from the major within the ten-month time period of the study.

We also captured retention behavior using the following two survey questions: (1) “Are you thinking about changing your major?” and (2) “How likely are you to change majors within the next year?” These questions were presented to students at the end of the fall quarter as well as at the end of the spring quarter. Overall, 27% and 34% of students, respectively, reported that they were considering a major change.

We examine the Likert scale response to the second question in consideration of our demographic data and enrollment in the EASE program. As shown in Table 6, we find that students placed in the EASE program reported lower values for this item, indicating that they were less likely than non-EASE students to change their intended major. Specifically, EASE students reported values 0.21 standard deviation units lower than those reported by their non-EASE counterparts after the fall quarter and 0.12 standard deviation units lower than those reported by their non-EASE counterparts at the end of the year, the latter of which is not statistically significant.

**Table 6:** Estimates for the impact of EASE on intent to change majors for the full sample and for student subgroups.

			(1)		(2)	
			End of fall quarter		End of first year	
Panel A.						
EASE			-0.207**	(0.105)	-0.12	(0.102)
Panel B.						
Gender						
	Full-sample estimates					
		EASE (Male)	-0.365*	(0.192)	-0.219	(0.231)
		EASE*Female	0.241	(0.228)	0.037	(0.273)
	Subsample estimates					
		Male (N = 263)	-0.365*	(0.193)	-0.219	(0.233)
		Female (N = 571)	-0.124	(0.123)	-0.182	(0.146)
Race						
	Full-sample estimates					
		EASE (White)	0.286	(0.231)	-0.399	(0.333)
		EASE*URM	-0.758***	(0.289)	0.052	(0.392)
		EASE*Asian	-0.458*	(0.278)	0.280	(0.379)
	Subsample estimates					
		White (N = 101)	0.286	(0.237)	-0.399	(0.343)
		URM (N = 284)	-0.472***	(0.173)	-0.347*	(0.207)
		Asian (N = 449)	-0.172	(0.153)	-0.119	(0.179)
First-generation status						
	Full-sample estimates					
		EASE	-0.230	(0.167)	-0.228	(0.172)
		EASE*First-gen status	0.070	(0.218)	0.004	(0.255)
	Subsample estimates					
		Continuing-gen status (N = 424)	-0.230	(0.167)	-0.228	(0.172)
		First-gen status (N = 410)	-0.160	(0.141)	-0.225	(0.188)
Low-income status						
	Full-sample estimates					
		EASE	-0.349**	(0.141)	-0.093	(0.168)
		EASE*Low-income status	0.382*	(0.203)	-0.149	(0.251)
	Subsample estimates					
		Low-income status = 0 (N = 494)	-0.349**	(0.141)	-0.093	(0.168)
		Low-income status = 1 (N = 340)	0.033	(0.146)	-0.242	(0.187)

**Table 6**. Robust standard errors in included in parentheses. All models include the following student controls: female, URM, Asian, first-generation status, low-income status, SAT Reading score, SAT Math score. The reference group is White. All items are standardized to have a mean of 0 and a standard deviation of 1. Higher values indicate more likely to change majors. Missing values have been adjusted using a dummy variable approach.

* p < 0.10

** p < 0.05

*** p < 0.01.

When examining specific demographic groups, we find that the impact of EASE on intent to leave the major is greatest for male and URM students, both groups of which reported values 0.37 and 0.47 standard deviation units lower, respectively, than their non-EASE counterparts when assessments were conducted at the end of students’ first term. It is important to note that the interaction term EASE*URM is negative and statistically significant. This indicates that EASE has the potential to reduce the White-URM student racial gap in regard to students’ intent to change majors.

### Limitations

The present study is not without limitations. Most notable among them is that the research design used is correlational, and we therefore cannot rule out the possibility that our results are subject to omitted variable bias. Given that participation in EASE is correlated with a number of demographic variables—EASE participants are more likely to earn lower standardized test scores, for example—we can assume that there are unobservable factors that are correlated with EASE participation and our outcome measures. For example, given that EASE participants are intentionally made aware of campus resources, they could very well be more likely to use them. One could imagine a scenario where EASE participants interact with the campus’ peer tutors more often than non-EASE participants. If true, the treatment effects reported in this study regarding course grades and GPA would be over-estimated.

In another example, given the limited number of variables available in the dataset, we may not have fully captured student ability and pre-college resources, both of which are correlated with a number of our outcome measures. This is particularly important since the characteristics associated with EASE participants indicate that they are more likely to come from lower quality high schools and from family backgrounds with less financial resources. Adding additional control variables, such as high school location and a refined measure of socio-economic status could provide a more precise, unbiased point estimate. It is important to note, however, that without these variables our point estimates are actually under-estimated.

Additionally, our findings associated with academic integration must be interpreted somewhat cautiously, as Cronbach’s alpha was only 0.60. This could indicate that the individual items in the scale have residual variance accounted for by different variables, other than academic integration. However, a student’s reported frequencies for “talking with faculty about academic matters,” “meeting with an academic advisor,” “meeting with a student mentor,” and “attending study groups outside of the classroom” could also be quite different from one another because students who frequently engage in at least one of these forms of academic help-seeking may not feel the need to engage in all of them. Although this would create low reliability for the scale, a higher average overall would still represent a greater amount of academic integration.

## Discussion

Our study has focused on a STEM learning community program, EASE, evaluating its impact on student cognitive and social-psychological outcomes. We have sought to investigate who benefits from learning community programs in order to better understand whether learning communities are a viable way to reduce both academic and motivation gaps often present in STEM disciplines. Overall, participation in the EASE program positively impacted both cognitive and social-psychological outcome measures. EASE designation is correlated with higher grades in Bio Sci 94, a key freshman level biology course, as well as with a nearly quarter-point boost in first-year cumulative GPA. Additionally, EASE students indicated that they experienced an improved sense of belonging and academic integration in addition to indicating that they were less likely to consider a change in major after participating in the program.

In examining the impact of the EASE learning community program on different groups of students, we find that students traditionally underrepresented in STEM exhibited the greatest gains regarding the study’s social-psychological measures. For example, females participating in EASE reported higher values for the sense of belonging measure than did non-EASE females. Further, female students, first-generation students, and students from low-income backgrounds all reported higher rates of engagement in behaviors indicative of academic integration relative to their non-EASE peers.

Surprisingly, however, the gains in social-psychological metrics do not correspond to disproportionate increases in academic outcomes for these same student populations. Although EASE status is associated with higher Bio Sci 94 course grades and first-year GPA, these effects tend to be greater for EASE students from more traditionally-represented demographics (males, White students, continuing-generation individuals, and non-low-income students). These results suggest that EASE does not reduce gender or racial achievement gaps in first-year biology courses. While much of the STEM education literature is focused on closing achievement gaps, we argue that the observed gains for the entire EASE population highlights the clear value of learning communities programs. The finding that EASE enrollment correlates with a nearly quarter point increase in first-year GPA relative to non-EASE participants means that an entire group of students may have increasing opportunities in STEM programs and careers. This may also imply however that many of the barriers to success for students underrepresented in STEM fields are also present in the EASE program. For example, while attempts were made to recruit underrepresented students to act as EASE mentors, they ultimately were predominately of Asian and White ethnicities. Thus, EASE students from underrepresented backgrounds may have had trouble connecting with their mentors or viewing them as representations of their own success. This would be similar to the impact of the lack of diversity in STEM faculty [53–54].

It is important to note that the discussed findings for cognitive and social-psychological measures point to the complex relationship between academic and social integration, as outlined in Fig 1. Academic and social integration are mutually reinforcing elements, and while we do not find evidence that these forces are operating concurrently—as evidenced by, for example, the idea that URM students experience disproportionate gains for a number of social-psychological measures but not for performance markers—prior research has found that treatment effects unfold over time [33,55]. As such, the social-psychological benefits experienced by students traditionally underrepresented in STEM may translate to positive long-run academic performance outcomes, such as strong grades earned in the second year of college or even major persistence. The consistent positive coefficients for the academic measures, although statistically insignificant, substantiate this conjecture. Our findings also suggest that examining long-run impacts will be important for fully understanding the ways in which learning communities improve student learning outcomes and the college experience as a whole.

We also find that the results concerning subgroup populations (Tables 3 and 5)—and for first-generation college students in particular—do not necessarily support Culture Mismatch Theory. As we note, first-generation students in EASE earned higher grades than their non-EASE counterparts (Table 3, column 2). These treatment effects, however, were not significantly different than continuing-generation students. In other words, EASE did not have a significant impact on reducing the socio-economic achievement gap.

First-generation college students also reported values for one of our outcome variables, academic and social concerns, in the opposite direction of what was expected. For this particular outcome, first-generation college students in EASE reported higher values, indicating that they were relatively more concerned that other students disliked them or unfairly evaluated their academic ability, as compared to non-EASE first-generation college students. One possible explanation could be the idea that academic preparation programs, such as EASE, might enhance feelings of stigmatization often felt among groups traditionally marginalized in college. Indeed, a separate study on EASE students found that both continuing- and first-generation students assigned to EASE felt somewhat stigmatized when learning of their assignment to the EASE program [56]. Initial feelings of stigmatization, in turn, were predictive of greater academic and social concerns during the school year. We do want to note that the point estimate for the variable academic and social concerns in our study is significant at the p < .10 level and the standard error is quite big; compared to other associations that we discuss in our paper, this one is relatively weak.

Lastly, even though the EASE program had no statistical impact on retention, we do find that it is positively correlated with students’ *intention* to stay in the bio sci major, which is particularly important given that this measure is an early indicator of engagement. Further, among the three race-oriented groups, this impact is most pronounced for URM students. URM students who participated in EASE reported values 0.47 standard deviation units lower than those reported by non-EASE URM students. This finding is particularly important given the national agenda to improve STEM outcomes for students least represented in STEM. A learning community certainly seems to have the potential to help URM students, in particular, progress through the STEM pipeline.

Our results also suggest that there might be a connection between the cognitive effects of participating in EASE and students’ *intent* to remain in the major, as outlined in Fig 1. For example, male students in EASE reported significantly lower values for the item measuring intent to leave the major than did non-EASE male students, whereas the difference for female students is not significant. The disproportionate impact for male students might be attributed to the large and significant impact that EASE had on male students’ academic performance. It may be the case that doing well academically makes students feel more confident about their future in the biological sciences.

Overall, our findings echo conclusions found in prior literature: learning communities benefit students both academically and social-psychologically. We add to this body of work by documenting the potential for learning communities to impact student learning and engagement within a specific field of study. Further, our focus on estimating impacts for particular student subgroups has resulted in evidence indicating that students respond to learning communities differently. Ideally, this evidence can help researchers and practitioners design programs tailored to meet different needs, thereby enhancing the ability of learning communities to positively impact the overall college experience.

## Supporting information

S1 File Items used for the social-psychological constructs evaluated by the survey instruments, the complete beginning of fall survey, and the complete end of fall and spring quarter surveys are presented in the supporting information. (DOCX)
