# Supplementary material for: Success with EASE: Who benefits from a STEM learning community?
Source: PLoS One. 2019 Mar 22;14(3):e0213827. doi: 10.1371/journal.pone.0213827 (PMC6430422; doi:10.1371/journal.pone.0213827)
Supplement: S1 File — (DOCX) [file pone.0213827.s001.docx]

**Supporting Information**

**Items Used For Each Social-Psychological Construct**

**Motivation - Interest Value (α=0.92)**

1. I’m really looking forward to learning more about Biology
2. Biology fascinates me
3. I think the field of Biology is very interesting

**Sense of Belonging in Biology (α=0.85)**

1. If I miss a Bio Sci class, I know students who I could get the notes from
2. I discuss events which happen outside of class with my Bio Sci classmates
3. I have developed personal relationships with other students in my Bio Sci classes
4. I feel comfortable volunteering ideas or opinions in my Bio Sci classes
5. I feel comfortable asking a question in my Bio Sci classes
6. I feel comfortable seeking help from my Bio Sci teachers before or after class
7. I feel comfortable asking my Bio Sci teachers for help if I do not understand course-related material
8. I feel comfortable asking my Bio Sci teachers for help with a personal problem

**Academic and Social Concerns (α=0.74)**

1. In college, I sometimes worry that people will dislike me
2. In college, I worry that people will think I’m unintelligent if I do poorly
3. In college, I often get nervous and worried when I talk to people

**Growth Mindset of Intelligence (α=0.90)**

1. You have a certain amount of intelligence, and you really can't do much to change it (reverse coded)
2. Your intelligence is something about you that you can't change very much (reverse coded)
3. You can learn new things, but you can't really change your basic intelligence (reverse coded)

**Academic Integration (α=0.60)**

1. Talk with faculty about academic matters, outside of class time (including e-mail)
2. Meet with an academic advisor concerning academic plans
3. Meet with a student mentor concerning academic plans (Antleader, peer mentor, EASE, etc.)
4. Attend study groups outside of the classroom
5. Have informal or social contacts with faculty members outside of classrooms and offices

**Beginning of Fall Quarter Survey**

**Introduction**

**Purpose:** You are invited to take this survey because the Ayala School of Biological Sciences values **your honest feedback about the major**. Sharing your thoughts and opinions will allow the School to better understand your experience of being a Bio Sci major, so that they may improve it for future students. The following questions ask you about your thoughts and opinions. **There are no right or wrong answers**.

**Confidentiality:** Your individual **answers will not be shared with any of your instructors**. If you have any questions or concerns about this survey, please contact the survey coordinators, Di Xu (dix3@uci.edu) or Peter McPartlan (pmcpartl@uci.edu).

**Compensation:** You will receive **course credit** for completing this survey.

______________________

**YOUR ATTITUDES ABOUT BEING A BIOLOGICAL SCIENCES MAJOR**

______________________

**Directions: Please indicate how true each statement is for you.**

*All items in this section on a scale from (1) Not at all true … (7) Very True*

**I. Desire to Contribute to Society**

1. I want to study biology because I want to make a contribution to society (desconw1)

**II. Confidence about Performance**

1. I am confident I will do well as a Bio Sci major (conf1w1)
2. I expect to get a good GPA as a Bio Sci major (conf2w1)

**III. Interest in Biology**

1. I’m really looking forward to learning more about biology (int1w1)
2. Biology fascinates me (int2w1)
3. I think the field of biology is very interesting (int3w1)
4. To be honest, I just don’t find biology interesting (reversed) (int4w1)

**IV. Perceived Utility Value**

1. How useful will knowledge of Bio Sci courses for your future? (util1w1)
2. I think what we are learning in Bio Sci courses is important (util2w1)
3. The material we are studying as Bio Sci majors is useful to know (util3w1)

**V. Attainment Value (Importance to identity)**

1. Being someone who is knowledgeable about biology is important to me (att1w1)
2. I feel that, to me, being good at solving problems which involve biology is important (att2w1)
3. Being knowledgeable about biology is an important part of who I am (att3w1)

**VI. Perceived Cost of being a Biology Major**

**Effort cost**

1. Considering what I want to do with my life, being a Bio Sci major is just not worth the effort. (coste1w1)
2. When I think about the hard work needed to get through my Bio Sci major, I am not sure that getting a Bio Sci degree is going to be worth it in the end. (coste2w1)

**Opportunity cost (family and friends)**

1. I worry about losing track of some valuable friendships if I am a Bio Sci major and my friends are not. (costo1w1)
2. I worry that being a Bio Sci major will take time away from other activities that I want to pursue. (costo2w1)
3. I’m concerned that my career goals in Biology will prevent me from being able to focus on marriage and family soon as I’d like to. (costo3w1)

**Psychological cost**

1. I’m concerned that I’m not a good enough student to do well as a Bio Sci major. (costp1w1)
2. I would be embarrassed if I found out that my work as a Bio Sci major was inferior to that of my peers. (costp2w1)
3. It frightens me that the courses required for the Bio Sci major are harder than courses required for other majors. (costp3w1)

_ _ _ _ _ _ _ _ _ _ _ _ _ _ _ _ _ _ _ _ _ _

**Directions: Here are a number of statements that may or may not describe your beliefs about learning biology. You are asked to rate each statement by selecting a number between 1 and 5 where the numbers mean the following:**

1. Strongly Disagree
2. Disagree
3. Neutral
4. Agree
5. Strongly Agree
6. Decline to answer

Choose one of the above five choices that **best expresses your feeling** about the statement. **If you don't understand a statement**, choose “6. Decline to answer.” **If you have no strong opinion**, choose “3. Neutral.”

**VI. Colorado Learning Attitudes about Science Survey (CLASS)**

***Real World Connections***

1. To understand biology, I sometimes think about my personal experiences and relate them to the topic being analyzed (clas1w1)
2. Learning biology changes my ideas about how the natural world works (clas2w1)
3. The subject of biology has little relation to what I experience in the real world (reversed) (clas3w1)

***Sense Making/ Effort***

1. There are times I think about or solve a biology question in more than one way to help my understanding (clas4w1)
2. When studying biology, I relate the important information to what I already know rather than just memorizing it the way it is presented (clas5w1)
3. When I am not pressed for time, I will continue to work on a biology problem until I understand why something works the way it does (clas6w1)
4. To learn biology, I only need to memorize facts and definitions (reversed) (clas7w1)

***Conceptual Connections/ Applied Conceptual Understanding***

1. After I study a topic in biology and feel that I understand it, I have difficulty applying that information to answer questions on the same topic (reversed) (clas8w1)
2. Knowledge in biology consists of many disconnected topics (reversed) (clas9w1)
3. If I don’t remember a particular approach needed for a question on an exam, there’s nothing much I can do (legally!) to come up with it (reversed) (clas10w1)
4. If I want to apply a method or idea used for understanding one biological problem to another problem, the problems must involve very similar situations (reversed) (clas11w1)

***Problem Solving Confidence***

1. If I get stuck on answering a biology question on my first try, I usually try to figure out a different way that works (clas12w1)
2. If I get stuck on a biology question, there is no chance I'll figure it out on my own (reversed) (clas13w1)

***Problem Solving Sophistication***

1. There is usually only one correct approach to solving a biology problem (reversed) (clas14w1)

_ _ _ _ _ _ _ _ _ _ _ _ _ _ _ _ _ _ _ _ _ _

**Directions: Please indicate how true each statement is for you.**

*All items in this section on a scale from (1) Not at all true … (7) Very True*

**VII. Sense of Belongingness in Biology**

**Peer Support**

1. If I miss a Bio Sci class, I know students who I could get the notes from (sbps1w1)
2. I discuss events which happen outside of class with my Bio Sci classmates (sbps2w1)
3. I have developed personal relationships with other students in my Bio Sci classes (sbps3w1)

**Perceived Classroom Comfort**

1. I feel comfortable volunteering ideas or opinions in my Bio Sci classes (sbcc1w1)
2. I feel comfortable asking a question in my Bio Sci classes (sbcc2w1)

**Perceived Isolation**

1. No one in my Bio Sci classes knows anything personal about me (sbiso1w1)
2. I rarely talk to other students in my Bio Sci classes(sbiso2w1)

**Faculty Support/ Comfort**

1. I feel comfortable seeking help from my Bio Sci teachers before or after class (sbfs1w1)
2. I feel comfortable asking my Bio Sci teachers for help if I do not understand course-related material (sbfs2w1)
3. I feel comfortable asking my Bio Sci teachers for help with a personal problem (sbfs3w1)

**Faculty Empathetic Understanding**

1. I feel that a Bio Sci faculty member would take the time to talk to me if I needed help (sbfu1w1)
2. I feel that a Bio Sci faculty member would be sensitive to my difficulties if I shared them (sbfu2w1)

**Extra Item**

1. I feel that I belong as a Bio Sci major (sbbiow1)

______________________

**YOUR ATTITUDES ABOUT BEING A COLLEGE STUDENT**

______________________

**Directions: Please indicate how true each statement is for you.**

*All items in this section on a scale from (1) Not at all true … (7) Very True*

**I. Belonging Uncertainty**

1. When something bad happens, I feel that maybe I don’t belong at UC Irvine (bu1w1)
2. I always feel that I belong at UCI (reversed) (bu2w1)

**II. Academic and Social Concerns**

1. In college, I sometimes worry that people will dislike me (asc1w1)
2. In college, I worry that people will think I’m unintelligent if I do poorly (asc2w1)
3. I am usually confident that others will have a good impression of my ability (reversed) (asc3w1)
4. In college, I often get nervous and worried when I talk to people (asc4w1)

_ _ _ _ _ _ _ _ _ _ _ _ _ _ _ _ _ _ _ _ _ _

**Directions: There are many reasons why people attend college. Please read the following list of possible motivating factors below. Please indicate how important each of the following reasons are for you.**

*All items in this section on a scale from (1) Not at all important … (7) Very important*

**IX. Independent & Interdependent Motives**

I am motivated to attend college because I want to…

1. become an independent thinker (independent) (iimot1w1)
2. learn more about my interests (independent) (iimot2w1)
3. prepare for a future career (independent) (iimot3w1)
4. expand my understanding of the world (independent) (iimot4w1)
5. expand my knowledge of the world (independent) (iimot5w1)
6. help my family out after I’m done with college (interdependent) (helping) (iimot6w1)
7. give back to my community (interdependent) (helping) (iimot7w1)
8. provide a better life for my own children (interdependent) (helping) (iimot8w1)
9. show that people with my background can do well (interdependent) (iimot9w1)
10. be a role model for people in my community (interdependent) (iimot10w1)

_ _ _ _ _ _ _ _ _ _ _ _ _ _ _ _ _ _ _ _ _ _

**Directions: Please indicate how much you agree with the following statements**

(1. Strongly disagree … 2. Disagree … 3. Mostly disagree … 4. Mostly agree … 5. Agree … 6. Strongly Agree)

**VIII. Growth Mindset of Intellgence**

1. You have a certain amount of intelligence, and you really can't do much to change it. (reversed) (gm1w1)
2. Your intelligence is something about you that you can't change very much. (reversed) (gm2w1)
3. You can learn new things, but you can't really change your basic intelligence. (reversed) (gm3w1)

______________________

**YOUR BACKGROUND**

______________________

1. Do you live with your parents or other family members during the academic year? (0. No...1. Yes) (lvwparw1)
2. Who are you most likely to talk to about course decisions and/or career plans? (1. Parent/guardian...2. Sibling...3. Other Family...4. Friend...5. Significant Other...6. Academic Advisor...7. Other...8. No one) (cartkw1)
3. If you selected “other” above, please list who else you would talk to (Open ended response) (cartkow1)
4. How often do you talk with this person about course decisions and/or career plans? (1. Daily...2. Weekly...3. Monthly...4. Rarely...5. Never) (cartkfw1)
5. Were you born in the United States? (0. No...1. Yes) (bornus)
6. Were your parents born in the United States?
   1. Mother (0. No...1. Yes) (bornusmo)
   2. Father (0. No...1. Yes) (bornusfa)
7. What language do you speak at home (e.g., with your parents)? (Open ended response) (homelang)
8. Was English your first language? (0. No...1. Yes) (engfirst)
9. At what age did you begin receiving most of your schooling in English? (engschl)
   1. I always received my schooling in English (1)
   2. age 2-4 (2)
   3. age 5-7 (3)
   4. age 8-10 (4)
   5. age 11-13 (5)
   6. age 14-17 (6)
   7. age 18-21 (7)
   8. after age 21 (8)
10. Was your mother (or guardian) educated in the United States? (0. No...1. Yes...9. Not Applicable) (edusmo)
11. Please mark the highest level of education your mother (or guardian) received: (hiedmo)
    1. Less than high school (1)
    2. High school (2)
    3. Some college (including associate’s degree or technical certification) (3)
    4. Bachelor’s degree (4)
    5. Graduate degree (e.g. master’s or doctoral degree) (5)
    6. Not applicable (9)
12. Was your father (or guardian) educated in the United States? (0. No...1. Yes...9. Not Applicable) (edusfa)
13. Please mark the highest level of education your father (or guardian) received: (hiedfa)
    1. Less than high school (1)
    2. High school (2)
    3. Some college (including associate’s degree or technical certification) (3)
    4. Bachelor’s degree (4)
    5. Graduate degree (e.g. master’s or doctoral degree) (5)
    6. Not applicable (9)
14. If you have adult siblings (18+), have they attended a 4-year college or university? (check all that apply) (sibcol)
    1. Attended and is still in school (1)
    2. Attended and graduated (2)
    3. Attended and dropped out (3)
    4. None of my adult siblings have attended a 4-year college/university (4)
    5. I do not have adult siblings (9)

INSTRUCTIONS: “Is there anything you would like to tell us?” (Open-ended) (extraqw1)

*~ END SURVEY ~*

____________________________________________________________________________

**End of Fall/Spring Quarter Survey**

**Introduction**

**Purpose:** You are invited to take this survey because the Ayala School of Biological Sciences values **your honest feedback about the course**. Sharing your thoughts and opinions will allow the school to better understand your experience and improve the major. **Your answers will not be graded in any way.** We just ask that you answer each question honestly. There are no right or wrong answers.

**Confidentiality:** Your individual **answers will not be shared with any of your instructors, TAs, or peers**. If you have any questions or concerns about this survey, please contact the survey coordinators, Di Xu ([dix3@uci.edu](mailto:dix3@uci.edu)) or Peter McPartlan ([pmcpartl@uci.edu](mailto:pmcpartl@uci.edu)).

**Credit:** You will receive **course credit** for completing this survey.

______________________

**YOUR ATTITUDES ABOUT BEING A BIOLOGICAL SCIENCES MAJOR**

______________________

**I. Desire to Contribute to Society**

**Instructions:** Please indicate how true each statement is for you.

(1. Not at all true … 7. Very true)

1. I want to study Biology because I want to make a contribution to society (desconw2)

______________________

**II. Confidence about Performance**

**Instructions:** Please indicate how true each statement is for you.

(1. Not at all true … 7. Very true)

1. I am confident I will do well as a Bio Sci major (conf1w2)
2. I expect to get a good GPA as a Bio Sci major (conf2w2)

______________________

**III. Interest in Biology**

**Instructions:** Please indicate how true each statement is for you.

(1. Not at all true … 7. Very true)

1. I’m really looking forward to learning more about Biology (int1w2)
2. Biology fascinates me (int2w2)
3. I think the field of Biology is very interesting (int3w2)
4. To be honest, I just don’t find biology interesting (reversed) (int4w2)

______________________

**IV. Perceived Utility Value**

**Instructions:** Please indicate how true each statement is for you.

(1. Not at all true … 7. Very true)

1. Bio Sci courses are important to my future (util1w2)
2. I think what we are learning in Bio Sci is important (util2w2)
3. The material we are studying as Bio Sci majors is useful to know (util3w2)

______________________

**V. Attainment Value (Importance to identity)**

**Instructions:** Please indicate how true each statement is for you.

(1. Not at all true … 7. Very true)

1. Being someone who is knowledgeable about Biology is important to me (att1w2)
2. I feel that, to me, being good at solving problems which involve Biology is important (att2w2)
3. Being knowledgeable about Biology is an important part of who I am (att3w2)

______________________

**VI. Perceived Cost of being a Biology Major**

**Instructions:** Please indicate how true each statement is for you.

(1. Not at all true … 7. Very true)

**Effort cost**

1. Considering what I want to do with my life, being a Bio Sci major is just not worth the effort (coste1w2)
2. When I think about the hard work needed to get through my Bio Sci major, I am not sure that getting a Bio Sci degree is going to be worth it in the end (coste2w2)

**Opportunity cost (family and friends)**

1. I worry about losing track of some valuable friendships if I am a Bio Sci major and my friends are not (costo1w2)
2. I worry that being a Bio Sci major will take time away from other activities that I want to pursue (costo2w2)
3. I’m concerned that my career goals in Biology will prevent me from being able to focus on marriage and family soon as I’d like to (costo3w2)

**Psychological cost**

1. I’m concerned that I’m not a good enough student to do well as a Bio Sci major (costp1w2)
2. I would be embarrassed if I found out that my work as a Bio Sci major was inferior to that of my peers (costp2w2)
3. It frightens me that the courses required for the Bio Sci major are harder than courses required for other majors (costp3w2)

______________________

**VII. Colorado Learning Attitudes about Science Survey (CLASS)**

**Instructions:** Here are a number of statements that may or may not describe your beliefs about learning biology. You are asked to rate each statement by selecting a number between 1 and 5 where the numbers mean the following:

1. Strongly Disagree
2. Disagree
3. Neutral
4. Agree
5. Strongly Agree

Choose one of the above five choices that **best expresses your feeling** about the statement. **If you have no strong opinion**, choose “3. Neutral.” Again, your answers will not be graded in any way, so please answer all questions as best you can.

**Real World Connections**

1. To understand biology, I sometimes think about my personal experiences and relate them to the topic being analyzed (clas1w2)
2. Learning biology changes my ideas about how the natural world works (clas2w2)
3. The subject of biology has little relation to what I experience in the real world (reversed) (clas3w2)

**Sense Making/ Effort**

1. There are times I think about or solve a biology question in more than one way to help my understanding (clas4w2)
2. When studying biology, I relate the important information to what I already know rather than just memorizing it the way it is presented (clas5w2)
3. When I am not pressed for time, I will continue to work on a biology problem until I understand why something works the way it does (clas6w2)
4. To learn biology, I only need to memorize facts and definitions (reversed) (clas7w2)

**Conceptual Connections/ Applied Conceptual Understanding**

1. After I study a topic in biology and feel that I understand it, I have difficulty applying that information to answer questions on the same topic (reversed) (clas8w2)
2. Knowledge in biology consists of many disconnected topics (reversed) (clas9w2)
3. If I don’t remember a particular approach needed for a question on an exam, there’s nothing much I can do (legally!) to come up with it (reversed) (clas10w2)
4. If I want to apply a method or idea used for understanding one biological problem to another problem, the problems must involve very similar situations (reversed) (clas11w2)

**Problem Solving Confidence**

1. If I get stuck on answering a biology question on my first try, I usually try to figure out a different way that works (clas12w2)
2. If I get stuck on a biology question, there is no chance I'll figure it out on my own (reversed) (clas13w2)

**Problem Solving Sophistication**

1. There is usually only one correct approach to solving a biology problem (reversed) (clas14w2)

______________________

**VIII. Sense of Belongingness in Biology**

**Instructions:** Please indicate how true each statement is for you.

(1. Not at all true … 7. Very true)

**Peer Support**

1. If I miss a Bio Sci class, I know students who I could get the notes from (sbps1w2)
2. I discuss events which happen outside of class with my Bio Sci classmates (sbps2w2)
3. I have developed personal relationships with other students in my Bio Sci classes (sbps3w2)

**Perceived Classroom Comfort**

1. I feel comfortable volunteering ideas or opinions in my Bio Sci classes (sbcc1w2)
2. I feel comfortable asking a question in my Bio Sci classes (sbcc2w2)

**Perceived Isolation**

1. No one in my Bio Sci classes knows anything personal about me (sbiso1w2)
2. I rarely talk to other students in my Bio Sci classes (sbiso2w2)

**Faculty Support/ Comfort**

1. I feel comfortable seeking help from my Bio Sci teachers before or after class (sbfs1w2)
2. I feel comfortable asking my Bio Sci teachers for help if I do not understand course-related material (sbfs2w2)
3. I feel comfortable asking my Bio Sci teachers for help with a personal problem (sbfs3w2)

**Faculty Empathetic Understanding**

1. I feel that a Bio Sci faculty member would take the time to talk to me if I needed help (sbfu1w2)
2. I feel that a Bio Sci faculty member would be sensitive to my difficulties if I shared them (sbfu2w2)

**Extra Item**

1. I feel that I belong as a Bio Sci major (sbbiow2)

______________________

**YOUR ATTITUDES ABOUT BEING A COLLEGE STUDENT**

______________________

**I. Belonging Uncertainty**

**Instructions:** Please indicate how true each statement is for you.

(1. Not at all true … 7. Very true)

1. When something bad happens, I feel that maybe I don’t belong at UC Irvine (bu1w2)
2. I always feel that I belong at UCI (reversed) (bu2w2)

______________________

**II. Academic and Social Concerns**

**Instructions:** Please indicate how true each statement is for you.

(1. Not at all true … 7. Very true)

1. In college, I sometimes worry that people will dislike me (asc1w2)
2. In college, I worry that people will think I’m unintelligent if I do poorly (asc2w2)
3. I am usually confident that others will have a good impression of my ability (reversed) (asc3w2)
4. In college, I often get nervous and worried when I talk to people (asc4w2)

______________________

**III. Growth Mindset of Intelligence**

**Instructions:** Please indicate how much you agree with the following statements

(1. Strongly disagree … 2. Disagree … 3. Mostly disagree … 4. Mostly agree … 5. Agree … 6. Strongly Agree)

1. You have a certain amount of intelligence, and you really can't do much to change it. (reversed) (gm1w2)
2. Your intelligence is something about you that you can't change very much. (reversed) (gm2w2)
3. You can learn new things, but you can't really change your basic intelligence. (reversed) (gm3w2)

______________________

**IV. Academic & Social Integration (Belongingness)**

**Instructions:** For the following questions, we want to understand how you spent your time this quarter

About how many hours do you spend **in a typical 7-day week** doing each of the following this quarter?

(0=0 hours per week; 1=1-5; 2=6-10; 3=11-15; 4=16-20; 5=21-25; 6=26-20; 7=31+)

1. Preparing for classes (studying, reading, writing, homework, lab work, etc.) (asi1w2)
2. Working for pay **on campus** (asi2w2)
3. Working for pay **off campus** (asi3w2)
4. Participating in co-curricular activities (organizations, campus publications, student government, fraternity or sorority, intercollegiate or intramural sports, etc.) (asi4w2)
5. Relaxing and socializing (watching TV, partying, etc.) (asi5w2)
6. Providing care for dependents living with you (parents, children, spouse, etc.) (asi6w2)
7. Commuting to class (driving, walking, etc.) (asi7w2)

**Instructions:** Please indicate how often you did the following activities this quarter. Consider all of your classes and activities, not just those for this course.

(0=Never, 1=Once a month, 2=Twice a month, 3=Every week)

1. Talk with faculty about academic matters, outside of class time (including e-mail) (act1w2)
2. Meet with an academic advisor concerning academic plans (act2w2)
3. Meet with a student mentor concerning academic plans (Antleader, peer mentor, EASE, etc.) (act3w2)
4. Attend study groups outside of the classroom (act4w2)
5. Have informal or social contacts with faculty members outside of classrooms and offices (act5w2)

______________________

**YOUR FUTURE PLANS**

______________________

**I. Behavioral Intentions (Looking to the Future)**

1. Have your experiences this quarter made you more sure or less sure of your major? (1=More sure; 2=Less sure; 3=No effect) (surew2)
2. Please explain how this quarter has made you more sure or less sure (open ended) (sureow2)
3. Are you thinking about changing your major? (0=no; 1=yes) (chgmajw2)
4. How likely are you to change majors within the next year? (1. Not at all likely … 7. Very likely) (chglikw2)
5. **If you are thinking about changing majors**, which major or majors are you considering switching to? (short open-ended) (chgoptw2)
6. **If you are thinking about changing majors**, what are the main reasons you would make the switch? (open-ended) (chgrsnw2)

______________________

**Instructions:**  “Is there anything you would like to clarify about your answers to any of the questions in this survey?” (Open-ended) (extraqw2)

*~ END SURVEY ~*

____________________________________________________________________________
